# Incorporating sense of place into the management of social-ecological systems: The researchers’ perspectives

**DOI:** 10.1371/journal.pone.0308726

**Published:** 2024-09-13

**Authors:** Joe Duggan, Sarah Clement, Christopher Cvitanovic, Ingrid van Putten

**Affiliations:** 1 Fenner School of Environment and Society, Australian National University, Canberra, Australia; 2 Department of Pacific Affairs, Australian National University, Canberra, Australia; 3 School of Business, Australian Capital Territory, University of New South Wales, Canberra, Australia; 4 Centre for Marine Science and Innovation, University of New South Wales, Canberra, Australia; 5 Environment, CSIRO, Hobart, Tasmania, Australia; 6 Centre for Marine Socioecology, University of Tasmania, Tasmania, Australia; Kwame Nkrumah University of Science and Technology, GHANA

## Abstract

As the world around us changes, so too do the bonds that people have with their environment. These environmental bonds, or Senses of Place (SoP), are a key component of social-ecological systems (SESs). SoP has social, psychological and economic value, it impacts how people use and behave in an environment and how they respond to changes such as those caused by anthropogenic pressures. Despite this connection, the consideration and application of SoP in the management of SESs remains relatively under explored. This study takes the first step in addressing that gap by investigating the perceptions of researchers in the field. We achieve this by interviewing SoP researchers from around the globe to elucidate whether they see SoP as valuable for the management of SES. We also identify their perspectives on the barriers to, and enablers of, incorporating SoP into policy and management. Results show while researchers do see the value in incorporating SoP into policy, there are a range of barriers that impact this, most notably, the intangible nature of the phenomena and shortcomings of current governance systems. Results also identify a range of enablers that could improve the incorporation of SoP into policy–particularly through improved engagement and communication between stakeholders. These findings represent a first step in the formal inclusion of this social value into the management of SES.

## Introduction

Sense of Place (SoP) has undergone an evolution in how it is conceptualised over the last 50 years. Originating in human geography research of the 1970’s [1,2], SoP was seen as linear, with a fixed end point. That is, the more time one spent in a place, the stronger their sense of that place. Such early definitions, however, did not capture the great deal of underlying complexity and nuance associated with the phenomena, i.e. recognition that it can grow, shrink and evolve over time, driven by changes to either people or place [3–5], and the fact it can be both tangible and intangible at the same time as being broad and dynamic, yet context specific [6].

Recently there have been calls to pluralise the phenomenon, that is, to acknowledge that there can be multiple competing and coexisting senses of place formed by individuals and groups [7]. For example, recent research in the Faroe Islands identified there were clear differences in SoP between locals, tourists and tourism brokers that resulted in tensions around a growing tourism industry in the region [8]. For our purposes we incorporate Raymond et al.’s [9] pluralisation of the phenomenon at the same time as adopting the definition of Hausman et al. [10] that contends SoP “*embeds all dimensions of peoples’ perceptions and interpretations of the environment*, *such as attachment*, *identity or symbolic meaning*, *and has the potential to link social and ecological issues”*.

The link to social and ecological issues is crucial, there is extensive, and growing research that suggests SoP is a key element in the effective function of social-ecological systems (SES). SoP can impact how people feel and how they behave within an SES. For example, Cunsolo Willox et al. [3] showed that a change to place (through climate change) impacted study participants health and wellbeing by disrupting their ability to undertake traditional activities in local landscapes. While Faulkner et al. [11] showed that increased place attachment is linked to increased community resilience in two coastal communities in the UK. Others have found that SoP can be a predictor of pro-environmental behaviour [12]. However, this relationship is not always intuitive, nor does it operate in a single direction–for example, Marshall et al. [13] showed a negative correlation between SoP and adaptive capacity in Peanut farmers in QLD, and others have shown SoP can be linked to both support or opposition of natural resource management policies [7].

Given the above, it is clear SoP and SES are linked, and that their relationship is increasingly important in light of growing anthropogenic pressures that threaten planetary boundaries that provide a safe operating space for humanity [14,15]. This linkage has led to calls for increased consideration of SoP in policy, particularly in the management of SES [6,16,17]. It is important to note that ‘policy’ is a term whose meaning can vary between groups. For our purposes we seek to take a broad view as per recent, comparable work [18]. When using the term policy, we are referring to legislation, planning processes and management that seek to guide the governance of SESs [19,20].

There are several key ways that SoP could be effectively incorporated into policy, such as: including diverse SoPs in the creation of policy [21]; creating policies that protect SoP [22]; and including SoP as a metric or variable into models that inform policy [23]. Despite clear pathways for impact, and suggestions from SoP researchers that SoP can and should be applied to policy [17], the formal incorporation of SoP into policy for SES management is limited. Work is currently underway to explore the drivers for this gap. Specifically, to understand whether policy makers see SoP as important for managing SES, and what the barriers or enablers are to its inclusion [18]. But policy makers are only one half of the equation, indeed for any effective transfer of knowledge across the research-practice-policy interface, we must also understand the perspectives of the stakeholders capturing and generating knowledge [24]. Thus, the aim of this study is to elucidate the perspective of researchers working on SoP to understand (i) whether they see SoP as important for policy, (ii) what they see as the opportunities for SoP to have impact, and (iii) the barriers to, and enablers of, incorporating SoP into policy. We achieve this by interviewing a globally distributed group of SoP researchers that have experience in measuring SoP within SES.

## Methods

### Participant selection and data collection

A qualitative approach was adopted to address the research aims. This allowed for an in-depth understanding of research participants experiences [25] and for the capture of multiple framings of SoP [26]. This was important considering that previous work has identified multiple framings as a key feature of the phenomenon [27]. The target participants for this study were SoP scholars with experience in capturing or measuring SoP within SES. Participants were initially drawn from a pool of researchers who were identified in a recent scoping review [28], and from a recently published seminal book on SoP [9]. Snowball sampling was undertaken to ensure adequate coverage of the participant group [29].

While there is no universally accepted ‘best practice’ sample size when undertaking qualitative research, evidence suggests that theoretical saturation is commonly achieved after 12 interviews [30]. For example, Tuohy et al. [31], with a sample size of 14, elucidated the skills and attributes that are required for research funders and managers to be successful knowledge brokers at the interface of environmental science and policy to build capacity for evidence-informed decision-making. In this study we continued interviews until new data failed to provide new insights [25], reaching theoretical saturation at 17 interviews. Thus, given the diversity of participants and reaching theoretical saturation, we posit that our sample of 17 is sufficient for the purpose of our study.

An interview guide was drafted with semi-structured interview questions to ensure consistency between interviews. This was created by the authors after discussing the research aims (see below) and drawing out lines of questioning to clearly address those aims. The interview guide was then refined after feedback was received from the third author, who was the first participant interviewed (i.e., pilot participant). The full interview protocol can be found at Appendix 1. Where unexpected themes emerged throughout the interview process, the guide was modified (following consultation between the first two authors).

Interviews were conducted by the first author through video conferencing software and ranged in length from 30–75 minutes. Detailed notes were taken in real-time throughout and the interviews were also recorded (with participant consent, and in line with ethical approval). Audio recordings were professionally transcribed to ensure their accuracy for analysis purposes.

Ethical approval to undertake this research was granted from the Australian National University Human Ethics Committee (Ref: 2022/307). Participants Information Sheets (PIS) were provided to all participants prior to participation (Appendix 2) and formal written consent to participate was obtained. In accordance with the approval protocol, demographic characteristics of the participants cannot be disclosed. Recruitment of participants took place from 8/7/22 to 11/11/22. Relevant participant demographics can be found in Appendix 3.

### Data analysis

Interview transcripts were analysed using the software program NVivo 12. The analysis involved broad thematic coding against the research aims:

1. Is SoP relevant for policy?
2. What are the opportunities for SoP to have impact?
3. What are the barriers to incorporating SoP into policy?
4. What are the enablers for incorporating SoP into policy?

An inductive coding approach based on Grounded Theory Analysis was adopted [32], whereby direct statements were coded, allowing for participants perspectives to emerge naturally [33], before a second round of coding was conducted by two authors (JD and CC) where the in-vivo codes were grouped thematically to identify broad themes.

To ensure emergent themes were relevant, they were continually compared against the raw data as per previous research e.g. [24,34–36]. This process continued iteratively until the codes were grouped into thematic hierarchies. Throughout the coding process, the number of interviews each code was recorded in was captured and not the frequency with which a theme was coded across all interview transcripts. This approach was adopted as frequency is not indicative of the importance that participants attribute to the specific themes (following previous studies such as [37,38]).

### Methodological limitations

It is important to note that for this study, we are choosing to only interview researchers. This is not to say that researcher perspectives are more important than any other group, simply that they are a valuable starting point for exploring the applicability of SoP to policy. Future work must include perspectives of other stakeholder groups from policy makers (as is already underway [18]) to community groups, so that a broad range of ontological and epistemological perspectives can be captured [39,40].

The authors of this study are researchers with interests in SoP, SES and/or adjacent fields. Our experiences of SoP and SES research originates mainly from Australia, Europe, North America, and the Pacific. We acknowledge our positions and accept that they will influence both the design of the research and the coding and analysis process. We have sought to adopt reflexive practice throughout the data collection, analyses and reporting to acknowledge and account for these biases as much as possible.

## Results

The coding of the 17 Interviews resulted in 14 themes that were mapped against the research aims (Table 1). It is important to note, that the numbers in this table do not indicate the level of importance that participants ascribed to each theme. The numbers only represent the number of participants that discussed the theme. Analysis of interview transcripts showed that 94% of interviewees agreed that SoP was at least in some way relevant for policy (n = 16). The specific themes are discussed in detail in the following subsections.

**Table 1:** Different themes about sense of place that emerged from data analysis for each of the research aims and the number of participants that mentioned each theme in the interview. The themes that were mentioned most are shown at the top for each research aim.

Theme	No. of participants
*Is SoP relevant for Policy*?	**16**
*Opportunities for SoP to have impact*	**16**
Incorporating values and perspectives into design	8
Communication and engagement	6
Stewardship	5
Academic impact	4
Crises and emergencies	1
Personal impact outside of policy	1
*Barriers*	**17**
Institutional and structural barriers	12
SoP is intangible and complex	8
Lack of communication and engagement	5
Tangible barriers	3
*Enablers*	**15**
More and better engagement	9
Organisational drivers	9
Features that enable	5
Research	4

### i) Opportunities for SoP to have impact

The majority of participants (n = 16) identified opportunities for SoP to have impact, be it in policy or elsewhere. Within this, six sub-themes emerged (Table 1). Of these, the most common was an acknowledgement by participants that SoP can allow for diverse values and perspectives to be incorporated into policy design (n = 8), as highlighted through the following quote: *“…senses of the place helps inform land use*… *It also helps inform more prescriptive policy options*, *whether it be around identifying specific areas around conflict management*, *identifying areas that need to be conserved*, *restored*, *or indeed changing the nature of developments in certain areas to be more aligned with local perspectives” (ID3)*.

The next most common theme to emerge from the analysis was that communication and engagement were key to allow SoP to have impact on policy (n = 6). Analysis shows this could be achieved by greater efforts, by researchers, to communicate the importance of SoP, as highlighted by one participant: *“that is often done through skilful persuasion… good description*, *and using straightforward language*, *effective*, *powerful language*, *so that’s a writing*, *communication skill”* (ID 13). Results also show that the notion of SoP could, in and of itself, also be used as a tool in communication and engagement as articulated by another participant: *“I found that the concept of place for me*, *in terms that it’s power for impact*, *has to do with being able to negotiate and renegotiate meanings of an environment that can be institutionalised in ways that they’re anachronistic” (ID 10)*.

Following this, the third most commonly discussed theme (n = 5) was that the impact pathway for SoP may lie in stewardship–as one participant stated: *“I think the opportunities are kind of like the political one*, *it’s […] political*, *influencing a sufficiently large group of people to put pressure on local governments*, *regional governments*, *or on a larger scale*, *national governments*, *to think differently about what’s going on”* (ID 13). A less common theme that emerged referenced the opportunity for SoP to have impact outside of direct policy impact, with four participants referenced academic impact. One respondent referenced global challenges, like climate change as an opportunity, in the sense that SoP would be more needed in policy in a world where our places are changing. Another respondent noted that there were opportunities for impact at the personal level, with an understanding of SoP being able to positively impact the world view of individuals.

### ii) Barriers to including SoP in policy

All participants identified at least one barrier to including SoP in policy based on their experiences. Four main themes emerged, most common were institutional and structural barriers (n = 13). Within this theme, six participants identified that policy makers typically require quantitative or financial information, and the fact that SoP was often captured with qualitative data, or at least was difficult to capture quantitatively, was a barrier. This was highlighted by one participant as: “*in my experience*, *it’s often easier to*, *when you’re talking*, *especially across sections*, *between academia and public sector or private sector*, *is usually easier to communicate if you have numbers*, *if you have quantitative data to show that*, *if you increase*, *or spend this much money*, *or get this return*, *blah*, *blah*, *blah*, *communicating qualitative benefits or uses is usually trickier*. *So that’s probably one of the main barriers”* (ID 6). A less common emergent theme was that SoP was often extremely context specific, while policy needed to be made ‘for everyone’ (n = 2) articulated by one participant as: *“…organisations often want levers and tools that are sort of ubiquitous*, *and universal across space and time*, *if you like*. *Or that aggregate to the larger hole or something*, *and this [SoP] just doesn’t work very well that way”* (ID 4). Other themes that emerged within institutional and structural barriers were the differences in world view between resource managers and other communities, hierarchies within institutions, failures in governance systems, an innate opposition to change in the general public and the drive of development trumping other priorities in policy.

The next most common theme that emerged when exploring barriers was the inherent challenges in capturing and incorporating a largely intangible and complex phenomenon into policy (n = 8). Within this, the complexity revolved around the fact that SoP was a phenomenon that included multiple facets and presented differently in different individuals and groups (n = 5), highlighted by one participant as: *“…*[A barrier is deciding] *what is it about sense of place you want to be integrated into the policy cycle*, *right*? *Understanding people’s attachment to place*, *for example*, *in and of itself isn’t going to give you an answer to anything” (ID 8)*. This theme was elaborated on by another participant as: *“how do you justify taking someone’s sense of place away*? *How do you justify making a policy to avoid taking someone’s sense of place away when the neighbour might have a different perspective*?*” (ID5)*.

The intangibility was specifically referenced by four participants and is captured well in the following quote: *“That kind of thing can’t get translated into policy because for the masses*, *the masses are not one to take in ambiguity; it becomes hard to implement*, *it just is the type of knowledge that then feels wishy-washy” (ID 14)*. Other elements that emerged under this theme were the fact there has not been enough research on how to incorporate SoP into policy and the fact that researchers were not expecting SoP to be incorporated.

Challenges in communication and engagement within and between policy makers and researchers was the next most common barrier (n = 5). This was framed by one participant as follows: *“I think another barrier is not everyone thinks about their world* [and] *how they live through the lens of senses of place*. *So in many cases our values and meanings related to our connection to place are not preformed*, *and we need new education processes and other deliberative processes to form them*. *And so then the opportunity here are around learning and education to make sense of the world around us”(ID 3)*. The final theme that emerged when considering barriers, was tangible barriers, three participants referenced limitations to cost and time, as with the following quote: *“working with locals and locals’ ideas about places*. *That is a costly thing basically”* (ID 15).

### iii) Enablers that encourage or allow the inclusion of SoP in policy

Most participants (n = 15, 88% of participants) discussed enablers that could help to overcome the barriers outlined above, and facilitate the inclusion of SoP into policy. Four main themes emerged through the coding of interviews. The most commonly referenced was increased and improved engagement (n = 9). This largely revolved around increasing trust, networks and collaboration between stakeholders–as one participant put it: *“… an enabler would be involving people as part of the policy making process*. *So [*..*] that would help to include Sense of Place into […] people[s] needs and expectations and motivations to be stewards as well of the place*. *So stewards of the policy almost*, *and stewards of the place”* (ID2).

Another commonly discussed enabler was organisational drivers (n = 9). Participants referenced a range of drivers that already existed—like the fact that SoP is starting to be mainstreamed, or the fact that there are some fields in some regions, like urban planning in Denmark, are already incorporating Sense of Place. Interestingly, climate change was seen as an enabler in this respect, as highlighted in the following quote: *“The enabler*, *again*, *earlier*, *it was an opportunity*, *now it’s an enabler*, *the idea that the crisis is screaming very loud*. *So politicians know that they have to do something with that because it resonates with their honest will or because it’s convenient for them to win the election*, *whatever*, *but it’s an enabler” (ID1)*. Other organisational drivers that emerged were: embedding SoP into policy processes that are already endorsed and spending more time problem solving at the local scale.

The third theme underscored the features of SoP that lend itself to inclusion in policy, from its flexibility to its innate relatability (n = 5). This was well articulated by one participant as: *“[SoP] is fundamentally fairly relatable*. *It’s not a super abstract academic construct that you have to spend two hours explaining*. *Usually people can understand it fairly straightforwardly*, *so that helps*. *So if you can talk to a decision-maker directly*, *at least they can understand what you’re trying to do*. *It’s not too abstract” (ID6)*.

The final theme highlighted the potential for research in key areas that could enable SoP inclusion in policy. Several participants (n = 4) identified areas for future investigation from exploring non-monetary incentives, to understanding the interplay of SoP and power and to conducting research that can show the benefits of incorporating SoP into policy: *“Maybe if there can be studies that show the impact*, *immediate impact*, *and show the success then it might be*, *because I think people all over the world realise it’s important*, *but perhaps we cannot yet show what the difference is between acknowledging it and not acknowledging it” (ID16)*.

## Discussion

SoP has been recognised in the recent literature as a phenomenon worth exploring particularly because of its complex and multilayered relationship with SESs [41,42], at a time when those systems are facing a range of external pressure [43]. By conducting in-depth interviews with SoP researchers, we were able to show that they did see SoP as relevant to policy, but a range of barriers, particularly institutional and structural ones, often limit its inclusion in decision-making processes. Encouragingly, we also discovered that increased engagement between stakeholders was a key enabler that could encourage the inclusion of SoP in policy. These findings are explored fully below.

### i) Opportunities for SoP to have impact

A key finding from the interviews was that the majority of participants felt that SoP was relevant to policy. Researchers have acknowledged for some time that considering the social side of SESs, including; cultural values [44], community values and cultural ecosystem services [45], well-being [46] and the context of place [47] can lead to more wholistic, measured and effective management of SESs [48]. As one example, when such elements are incorporated into policy it can lead to the increased acceptance of that policy by key stakeholders [49], which could in turn lead to greater compliance with that policy. It is of little surprise then that SoP was seen as another phenomenon worth including. While it could be expected that this theme emerged simply because the participants had SoP research backgrounds and would undoubtedly want (or need) to do research that is policy relevant, the breadth of themes emerging around opportunities for impact and the broad range of enablers and barriers suggests that there is more to the story here, and mirrors previous calls for SoP to have greater impact on policy and practice [50]. If we accept that there are opportunities for SoP to have positive impact on policy, the next question is, *how*?

Participants suggested that using SoP to incorporate the values and perspectives of the groups that policy will eventually impact, into that policy, could strengthen the support and adherence to associated rules and legislation. Incorporating diverse values into policy is not a new idea, having been pioneered by Arnstein [51] who outlined levels of community involvement in urban planning. SES researchers have long recognised the need to integrate different voices, knowledge systems and actors in research and practice to build adaptive capacity and ensure system resilience [52,53], and have been amongst the researchers that have turned to participatory research to achieve this [54].

Using SoP as a tool to incorporate diverse voices and actors into policy design is certainly feasible and would be more achievable when adopting the contemporary conceptualisation of the phenomenon, that is by pluralising SoP and allowing for multiple, contested views [7], one can then, in theory, consider those diverse values in the creation of policy. While it is beyond the scope of this current research, it would be crucial to understand how multiple and contested views could be included into policy in a constructive way without increasing conflict [55].

The second most common theme raised by participants as an opportunity for SoP to have impact on policy was communication and engagement. This was framed via two key pathways; either through communication of the importance of SoP or using SoP as a tool in communication and engagement. This is particularly interesting considering that earlier research has identified that SoP is often viewed as a boundary object [6] that is a tool for knowledge sharing [56] and a way for stakeholders to share and compare meanings and understanding if places. Perhaps this then positions SoP as a possible tool to facilitate the effective inclusion of voices called for above. Although, further work is required to fully understand the exact mechanisms to achieve this.

Less common, but still interesting were the themes that emerged around stewardship. Within this, some researchers felt that incorporating SoP into policy would have impact by acknowledging stakeholders ownership and responsibility over a place and could hence lead to greater stewardship. This link between stewardship and SoP has been recognised in a range of SESs where SoP can motivate stewardship and vice versa [41]. For example, Murphy et al. [57] identified that caring for urban lakes in Bangalore, India reinforced place attachment and meanings of participants. The subsequent themes that emerged are less common again and more removed from direct policy impact and are thus beyond the scope of this current study to explore in depth.

### ii) Barriers to including SoP in policy

A range of themes emerged as barriers impacting the inclusion of SoP in policy. While these themes are often cited as causes of the science-policy gap [58], or challenges in knowledge exchange at the science policy interface [59], this study is the first to consider their implication specifically when considering how to include SoP into the management of SES.

The most common barrier emerging from the data was institutional and structural barriers. This is often cited as a limiting factor in similar areas of research. For example, in conservation a mismatch in approaches and priorities of research and policy groups has been recognised as a barrier in the implementation of research informed policy [60,61]. Within institutional and structural barriers, a sub theme emerged suggesting policy makers require numbers and clear quantitative data that can be related to financial implications, while researchers seek to capture the nuance and complexity of SoP through qualitative means. This contradiction has been acknowledged in a range of fields from education [62] to environmental change research [63]. This barrier could be originating from the belief that SoP is a context specific and dynamic phenomenon, and as such one that may lend itself to being measured through qualitative means [27]. However, this requires more research to fully understand, particularly when considering a recent review of the literature suggested that 40 percent of SoP studies adopted mixed methods, while 27 percent adopted purely quantitative methods [28], suggesting that quantitative data around SoP is indeed being captured. Regardless of the causes for this disconnect, addressing it will likely be more complicated than simply capturing more quantitative measurements of SoP. Research has shown that quantitative data can be misrepresented in policy [64] and policy makers are not necessarily making full use of available data when designing management plans [65], relying on other sources such as personal experience [66].

Another common barrier highlighted in this study revolved around poor communication between either side of the science-policy divide. Again, this has been referenced as a barrier to evidenced informed policy making across the sciences at large [67,68] as well as within specific fields such as environmental management [69] and conservation [70]. Researchers exist within a publish or perish culture and often have more urgent priorities than communicating their research to policy makers [71] or they may lack the required communication skills [70]. Regardless of the driver, it is clear this is a significant barrier and extensive research is underway in how to overcome it (see [37,59,72–74]). It is worth noting here that framing the issue as a binary could be seen as reductivism and an oversimplification of a complex issue. For our purposes, it is a concise articulation of a broad problem and going forward, it is possible that SoP, as a phenomenon rooted in social science and humanity, could be a valuable tool in altering the discourse around science for policy and actively contribute to breaking down that binary [75].

The intangibility of SoP has been recognised as a barrier to measurement in previous studies [6], but we would caution against seeing this as solely negative for the incorporation of SoP into SES management. Ecosystem service frameworks have long dealt with intangible concepts and sought to combine or contrast them with the tangible for policy outcomes [76,77], it is feasible that its incorporation into Ecosystem Service frameworks is a possible way forward for SoP [10]. There is a risk here though that incorporating SoP as one of many ecosystem services would result in minimising and oversimplifying the phenomenon, Magee et al. [78] provide an alternative, acknowledging the intangibility of SoP, but using quality of life survey data alongside house pricing and demographic statistics to measure changes in SoP following a natural disaster. While this only offers up a single SoP for an entire town and hence ignores the evolving conceptualisation of SoP, it is an area worth exploring more.

### iii) Enablers that encourage or allow the inclusion of SoP in policy

While previous research has sought to understand enablers at the science-policy interface, this study was the first to do so explicitly for SoP. More and better engagement between stakeholders, be they researchers, policy makers or the groups the policy stands to impact, was an often-cited enabler as has been referenced in the environmental policy-practice literature broadly [63,70,79]. The emergence of this enabler is unsurprising considering a lack of communication was identified as a barrier. Within this though, there is cause for optimism—researchers identified that increased engagement represented more than just increased communication or improved understanding of SoP for policy makers. That is, there was a recognition that improved trust, networks and collaboration could allow for the inclusion of SoP into policy. This marks a change in direction from traditional views of science communication as a one-way exchange from researchers to end users–the deficit model [80], towards two-way knowledge-exchange and ideally, knowledge co-production [36,74,81]. Certainly, this is a fruitful area for future research, particularly around the value that a dedicated actor could provide in terms of knowledge brokerage [82,83]. As a starting point Reed et al., [72] provide a range of principles to support this form of engagement in environmental management from setting appropriate goals at the outset, building long term trust and incorporating reflexivity.

Perhaps the most interesting organisational driver to emerge from the data was climate change. This is particularly interesting because climate change is well known for eliciting a range of negative responses in researchers [84,85]. The fact that some researchers are identifying benefits is a macabre silver lining if nothing else. It also highlights that as the world is changing around us there will be new and novel ways and opportunities to incorporate SoP into policy. To leverage this enabler further, work should be carried out exploring the case studies where SoP is effectively being incorporated into policy (e.g. [16]).

Results also show that there were features of SoP that emerged as enablers, features that were at least in part related to barriers–that is, the complexity and intangibility of SoP was seen as a barrier, but its flexibility and relatability was seen as an enabler. While at first this may seem like a contradiction, the distinction may lie in what the end point of the phenomenon is–a policy document or an individual’s worldview. It could be argued that policy is designed to be definitive, clear and without ambiguity [86] so incorporating something intangible and complex is inherently difficult. Whereas humans are made up of infinite diverse experiences that influence their ontologies and epistemologies, as such, it is possible that a complex and multi-faceted phenomenon like SoP [6], offers up a range of faces and interpretations that can tie into any individual’s unique way of knowing or being.

This contrast clearly requires more research, as it could also be argued that in the area of SES management, a large amount of policy is not clear and is often left intentionally vague. Broad terms like ‘resilience’ are included in policy with increasing frequency. Terms such as these often have multiple contrasting definitions and are often used to describe largely intangible phenomena [87]. If they are able to be included in policy, then with the appropriate framing and context, perhaps SoP can as well.

## Limitations and future research

Fully understanding SoP is crucial during this period of global change and dynamic disruption, so as to ensure the resilience of SESs into the future. While this could focus on a range of possible areas–from the application of SoP in SES research [41] or the application of SoP as a tool in knowledge brokerage [6,88] we are choosing to begin with an exploration of the applicability of SoP in the management of SES and within this focussing on researchers as a starting point. While this research marks an important starting point, it will also become a valuable reference as the views of other groups are explored (e.g. [18]). There are however a number of limitations and future research directions that must be noted.

Firstly, while steps were taken to ensure that participants were diverse, at least in their geographic location, the field at large is biased towards research conducted in and focussing on, the global north (see [17]). As such the results do not consider traditional ways of being and knowing [40,89], this marks a priority area for future research, particularly when considering the importance of including diverse ontologies and epistemologies in effective co-production of management plans and practices [89].

While several broad themes were highlighted as opportunities for SoP to have impact on environmental policy, the exact tool, or approach that would allow for this is still unclear. The context-specific nature and the broad range of conceptualisations for the phenomenon [27], mean that a one size fits all approach is unlikely to be successful. But that is not to say that policies can not be developed centrally and adapted locally, as has been shown most recently in climate adaptation [90]. Certainly, a basic heuristic, or a toolkit for researchers and policy makers alike could prove helpful [91], and certainly there are lessons to be learnt from fields like urban design who are more frequently putting SoP at the forefront of policy [92], but this must be undertaken with an awareness of the barriers and enablers outlined above.

## Conclusion

In this study we have used in-depth qualitative interviews with SoP researchers to explore the opportunities for SoP to impact on policy and to understand the barriers and enablers to this happening effectively. We have identified that while researchers feel SoP is important for policy, there are a range of barriers limiting its inclusion. Many of these barriers are the same ones we see at the science-policy interface more broadly from institutional and structural barriers to poor communication and engagement and resource constraints. Outside of these typical barriers, it appears that the complex and intangible nature of SoP could be an additional hindrance. Despite this there are key enablers that stand to improve its incorporation into policy, from increased engagement to organisational drivers and the phenomenon’s inherent relatability. While further research is still required to fully understand how to maximise the utility of SoP for environmental sustainability, it is hoped that these findings represent a first step in a more nuanced understanding of how SoP can inform policy.

## Supporting information

S1 Appendix Interview protocol. (DOCX)

S2 Appendix Participant information sheet. (DOCX)

S3 Appendix Demographic information of study participants. (DOCX)
