# Supplementary material for: Incorporating sense of place into the management of social-ecological systems: The researchers’ perspectives
Source: PLoS One. 2024 Sep 13;19(9):e0308726. doi: 10.1371/journal.pone.0308726 (PMC11398684; doi:10.1371/journal.pone.0308726)
Supplement: S1 Appendix — (DOCX) [file pone.0308726.s001.docx]

**Interview Protocol**

**Preamble**

Welcome and thanks, talk about myself and my research, explain why I want to interview them.

**What is your understanding of SoP?**

**What is your experience of SoP?**

Prompt: Can you give me an example of where sense of place has mattered to you

**Definition of SoP:**

The emotional bond that people have with a ‘place’

SoP ‘embeds all dimensions of peoples’ perceptions and interpretations of the environment, such as attachment, identity or symbolic meaning, and has the potential to link social and ecological issues’.

1. **Introductory questions**
   1. Tell me about yourself
   2. Tell me about your Sense of Place (SoP) Research
2. **Understanding the participants motivations and drivers for studying SoP**
   1. Why do you think studying SoP is important?

*Prompt: Why do you study SoP?*

1. **Understanding how they measure SoP and why**
   1. What methods have you used for measuring SoP before?
   2. Why did you use those methods? Are there any other methods you considered?
   3. Of the methods you did use, what were the advantages and disadvantages?

*Prompt: internal/external/personally/other*

1. **Opportunities for improvement**
   1. In a perfect world (if resources, time, money were not a limiting factor) how would you chose to measure SoP?
   2. What are the barriers that have stopped you from doing this?

*Prompt: Can you give examples?*

- 1. Can you think of specific ways to overcome these barriers?

1. **Opportunities for impact**
   1. What are the opportunities for turning research into impact?
   2. What are the opportunities to apply research for positive outcomes?
   3. Do you think SoP is important for policy?
   4. What are the barriers in including SoP in policy considerations?

*Prompt: internal/external/personally/other.*

- 1. What are the enablers in including SoP in policy considerations?

*Prompt: internal/external/personally/other*

1. **Conclusion**
   1. Now that we have completed the formal component of the interview, are there other important issues that were not covered by our questions, or other relevant insights that you would like to share based on you experience?
   2. And finally, considering the topic of the study, and the questions I’ve just asked you, who are three other people that you think I should interview in this research?

*Thanks and next steps*
