# Supplementary material for: Incorporating sense of place into the management of social-ecological systems: The researchers’ perspectives
Source: PLoS One. 2024 Sep 13;19(9):e0308726. doi: 10.1371/journal.pone.0308726 (PMC11398684; doi:10.1371/journal.pone.0308726)
Supplement: S2 Appendix — (DOCX) [file pone.0308726.s002.docx]

**Participant Information Sheet**

**Project Title**

Improving the application of Sense of Place to resource management

**General Outline of the Project:**

- **Purpose and methods:** The aim of this project is to address gaps in the measurement and application of Sense of Place for resource management. This will include the exploration the barriers and enablers to SoP measurement as well as the motivations and drivers for studying SoP. To address these questions, this study will use in-depth qualitative interviews of academic researchers with experience in applied SoP research.
- **Use of Data and Feedback:** We will use the data to contribute to scholarly publications/conferences.
- **Research team:**
  - Mr Joe Duggan – Primary Investigator
  - Dr Christopher Cvitanovic – Co-investigator
  - Dr Ingrid van Putten – Co-investigator

**Participant Involvement:**

- **Voluntary Participation & Withdrawal:** We invite you to participate in this research given your experience as a researcher of Sense of Place. You do not have to be involved in this research unless you want to, and you can pull out of the research if you change your mind without telling us why. If you do withdraw, we will not use the information you have provided at any time prior to finalisation of the study.
- **What does participation in the research entail?** You have been invited to participate because of your experience as a researcher of Sense of Place. Your participation will include an interview of approximately 45-60 minutes over zoom at a time of your convenience. Should you give written consent (please see consent form attached to this email), interviews will be audio recorded and professionally transcribed to ensure their accuracy. As such you will not be required to review your interview transcript, but can request to do so if you would like.
- **Risks:** Participation in this study should involve no risks beyond those of everyday living. During all activities, you are under no obligation to answer any questions with which you are not comfortable, or provide any information you would prefer to withhold. Even if you agree to participant, you are also free to withdraw from the study at any point up until the results are finalised and published without having to provide an explanation. There is no penalty for refusing to participate in this study, withdrawing your participation at any point up until the project is finalised, or not answering specific questions. If, for some reason, participation in this project does cause you distress, we encourage you to email the contacts named at the end of this form who will assist you with identifying a suitable support avenue (e.g. counselling).
- **Benefits:** We expect participation will be enjoyable and meaningful, and will provide opportunities for participants to contribute to how SoP can be measured and applied. Through the publication of the outcomes of the project in academic journals, we will share the lessons from the project with the wider research community. To this end, the final published work will be shared with participants following finalisation of the project. However, there is no other direct benefit to participants from participating in the project.

**Confidentiality:**

Confidentiality will be protected as far as the law allows. To ensure confidentially, only the research team (named above) will have access to the raw data, and all of your responses to the interview will be processed so that unauthorised users cannot access them, in accordance with the Australian Human Research Ethics National Statement. To this end, we will not include any personal information in reports or papers and will seek to ensure that unless specific permission is obtained, comments or findings are presented in a way that is not individually identifiable. Although steps will be taken to protect the identity of the participants, there is a risk that others may guess the source of information. Therefore, participants should avoid disclosing information that is confidential, defamatory or that could otherwise harm their interests.

**Privacy Notice:**

In collecting your personal information within this research, the ANU must comply with the Privacy Act 1988. The ANU Privacy Policy is available at <https://policies.anu.edu.au/ppl/document/ANUP_010007> and it contains information about how a person can:

- Access or seek correction to their personal information;
- Complain about a breach of an Australian Privacy Principle by ANU, and how ANU will handle the complaint.

**Data Storage:**

- **Where and how long:** Data (including notes and analysis materials) will be stored for a minimum of five years following publication on ANU secure servers. De-identified hard copy materials will be stored in a locked filing cabinet accessible only to the research team. After five years, all raw data will be destroyed (i.e. computer files completely deleted, hard copy notes shredded, etc.).
- **Handling of data following the required storage period:** Following all use of the data (i.e. reporting on outcomes of the project and contributing to academic publications), data will be retained by the ANU on secure servers.

**Queries and Concerns:**

- **Contact Details for More Information:** Enquiries about the project can be directed to Mr Joe Duggan via [Joe.Duggan@anu.edu.au](mailto:Joe.Duggan@anu.edu.au) or +61 447 143 433.

**Ethics Committee Clearance:**

The ethical aspects of this research have been approved by the ANU Human Research Ethics Committee (Protocol 2022/307). If you have any concerns or complaints about how this research has been conducted, please contact:

Ethics Manager
The ANU Human Research Ethics Committee
The Australian National University
Telephone: +61 2 6125 3427
Email: [Human.Ethics.Officer@anu.edu.au](mailto:Human.Ethics.Officer@anu.edu.au)

**Thank you for considering to participate in this study.**
