# Supplementary material for: Incorporating sense of place into the management of social-ecological systems: The researchers’ perspectives
Source: PLoS One. 2024 Sep 13;19(9):e0308726. doi: 10.1371/journal.pone.0308726 (PMC11398684; doi:10.1371/journal.pone.0308726)
Supplement: S3 Appendix — (DOCX) [file pone.0308726.s003.docx]

**Demographic information of study participants**

*Gender*

| **Participant Gender** | n= |
| --- | --- |
| Female | 9 |
| Male | 8 |

*Location*

| **Country of Research Institute** | n= |
| --- | --- |
| United States | 5 |
| Finland | 5 |
| South Africa | 2 |
| Australia | 1 |
| Germany | 1 |
| Sweden | 1 |
| Canada | 1 |
| Denmark | 1 |
